# A new index for frequency stability assessment in Low-Inertia Power Systems

**DOI:** 10.1371/journal.pone.0340648

**Published:** 2026-01-07

**Authors:** Sishi Qin, Sim Sy Yi, Chao Zhang, Huaying Zhang

**Affiliations:** 1 School of Power Engineering, Guangxi Electrical Polytechnic Institute, Nanning, China; 2 Faculty of Electrical and Electronic Engineering, University Tun Hussein Onn Malaysia, Batu Pahat, Johor, Malaysia; 3 Energy Planning Research Center, China Energy Engineering Group, Guangxi Electric Power Design Institute Co., Ltd., Nanning, China; Aalto University, FINLAND

## Abstract

As grid-forming converters (GFM) and grid-following converters (GFL) continue to be integrated into low-inertia power systems in place of traditional synchronous generators, the characteristics and forms of system inertia have undergone significant transformation. This evolution poses significant challenges to conventional inertia response mechanisms and analytical methodologies. To address these challenges, this study proposes a novel grid index frequency stability margin (FSM) from the perspective of frequency stability, encompassing its definition, quantitative evaluation, and practical applications. This paper first introduces the mathematical foundations and operational definitions of the FSM. It then systematically investigates the factors influencing FSM and presents a comprehensive mathematical model specifically developed for low-inertia power systems. The FSM calculation method based on aggregated system modelling was developed, followed by the derivation of a simplified estimation approach suitable for practical engineering applications. The effectiveness of the FSM in analyzing the frequency stability of low-inertia grids was validated through case studies based on provincial-level power grid data from China and a modified IEEE 39-bus system. The findings establish a theoretical framework for optimizing the planning and development of new energy power plants, as well as for formulating grid operation control strategies. This framework offers essential guidance to ensure the secure and stable operation of low-inertia power systems.

## 1 Introduction

Integrating renewable energy into power grids necessitates converter-interfaced generation (CIG), which is becoming increasingly central to power systems as renewable energy penetration grows. Current converter technologies primarily consist of the grid-forming converter (GFM) and grid-following converter (GFL). In practical engineering, the vast majority of GFL operate in constant power control mode and do not provide inertial support to the system. In this study, the term “GFL” specifically denotes those units that do not deliver system inertia. Certain improved GFL implementations can deliver limited inertial support via additional virtual inertia control strategies [1–3]; however, their response characteristics fundamentally differ from those of the GFM. While it improves system performance, its response speed, support depth, and stability are constrained by the measurement accuracy and dynamic response capabilities of the phase-locked loop (PLL). By contrast, the GFM can provide virtual inertia with adjustable equivalent inertia time constants. The large-scale integration of CIG alters the inertia characteristics of power systems, thereby posing new challenges to conventional analytical methods.

In modern frequency stability analysis, the equivalent inertia time constant and damping coefficient serve as fundamental parameters. Contemporary research employs phasor measurement units (PMUs) to monitor these parameters and has developed various analytical methods, including impedance analysis [4], data-driven approaches [5], system identification [6], and modal analysis [7]. These approaches have collectively established a theoretical foundation for frequency stability assessment in low-inertia power systems.

Two primary paradigms exist for characterizing the equivalent inertia time constant which are the traditional time-dimensional index and modern energy-dimensional representations. Zhai et al. [8] employs time-dimensional inertia constants to demonstrate decreasing grid inertia trends, concluding that frequency stability is progressively deteriorating. In conventional power grids, the equivalent inertia time constant is decoupled from the grid capacity since rotating generator sets inherently possess rotational inertia. However, in low-inertia power systems, where specific power sources are incapable of providing inertia, the equivalent inertia time constant becomes dependent on the grid capacity. Consequently, a lower inertia time constant does not necessarily imply a degradation of disturbance resistance; instead, an increase in system capacity can maintain or even enhance stability despite a reduction in the inertia constant [9].

Energy-dimensional inertia index is widely adopted in international standards and operational guidelines because it provides a direct and intuitive basis for grid security assessment, as reflected in requirements such as those specified by AEMO. For instance, the Australian Energy Market Operator specifies 6000 MWs as the minimum inertia requirement and 4400 MWs as the operational safety threshold [10]. The extensive adoption of the GFL, which operates under constant power output control, has significantly weakened the system’s inertia support capability. As a result, disturbances may activate Rate-of-Change-of-Frequency (RoCoF) and frequency threshold protection mechanisms. Consequently, some current research has focused on the equivalent inertia time constant (represented in kinetic energy units) under system dynamic frequency constraints encompassing RoCoF and rated frequency deviations. A recent study [11] identified post-fault inertia requirements exceeding 130 GVA. s for the UK grids, based on predefined RoCoF limits and credible contingency scenarios. García-Ruiz et al. [12] proposed an integrated assessment method that combines frequency deviation and RoCoF constraints, which ENTSO-E has adopted for evaluating minimum inertia. Additionally, Zang et al. [13] have established minimum safety inertia requirements under similar constraints. The energy-dimensional index only reflects the total kinetic energy of the system. Still, it does not encompass the dynamics of the frequency regulation process (such as FCAS response speed and damping characteristics).

To address this limitation, this study introduces a new index, the Frequency Stability Margin (FSM), under system dynamic frequency constraints. The advantage of the FSM lies in its simultaneous consideration of inertial kinetic energy and frequency regulation capability, thereby enabling a more comprehensive assessment of the maximum tolerable power deficit and providing a reference for analyzing grid frequency stability. This paper introduces the Frequency Stability Model (FSM) as a method for effectively analyzing frequency stability in low-inertia power grids. It details computation methodologies for the FSM, which include both an aggregated model-based approach and a Phasor Measurement Unit (PMU)-based estimation method. The FSM explicitly quantifies the maximum tolerable power deficit under both frequency and RoCoF constraints while enabling vulnerability identification for planned grids across multiple operational scenarios. This facilitates the determination of secure renewable energy penetration levels and control strategies.

## 2 The FSM concept

When the power system frequency reaches its stable upper limit, it can be regulated through measures such as generator tripping and load increase to achieve frequency restoration at a relatively low cost. In contrast, when the frequency drops to the stable lower limit, restoring frequency requires increasing the system output power. This process requires a comprehensive consideration of factors such as whether the system’s inertia and reserve capacity are sufficient, as well as the economic costs associated with increasing power output. Consequently, higher requirements are imposed on grid planning and operational strategies. This paper examines the lower frequency stability limit in power systems and subsequently proposes the Frequency Stability Margin (FSM) as a new analytical metric.

The Frequency Stability Margin (FSM) is defined as the maximum tolerable power deficit at the grid nodes that simultaneously satisfies: 1) power flow equations and operational security constraints imposed by equipment limitations, 2) system frequency control actions within the inertial response timeframe, and 3) frequency stability requirements. Assuming that Points 1 and 2 are satisfied, this paper primarily addresses Point 3.

Thus, the FSM quantitatively defines the tolerable power deficit that guarantees that both RoCoF and the rated frequency deviation persistently adhere to operational thresholds throughout the inertial response phase and the primary frequency regulation phase, as formulated below:


$$P_{f}(t)=min(P_{\Delta f},P_{rocof})$$ (1)

where $P_{\Delta f}$ represents the FSM under frequency deviation constraint, and $P_{rocof}$ represents the FSM under RoCoF constraint. The FSM integrates M_sys_, frequency deviation constraint, the RoCoF constraint, and regulation capacity through equation (1). This approach simultaneously covers both the inertial response and the primary frequency regulation processes. $P_{rocof}$ is derived from equation (12), and $P_{\Delta f}$ can be calculated using the two methods proposed in this paper, which are the aggregated model and a PMU-based approach.

According to Gu et al. [14], the RoCoF is mainly determined by the system’s equivalent inertia time constant. In contrast, the lowest point of frequency dip (i.e., the maximum frequency deviation) is jointly determined by the equivalent inertia time constant and frequency control ancillary services (FCAS). FCAS acts similarly to a remedial mechanism after “braking.” It activates after a fault occurs and gradually offsets the power deficit through dynamic response, thereby preventing further frequency decline and promoting recovery. The response performance of FCAS is closely related to factors such as the reserve capacity of generating units and the frequency regulation coefficient. Therefore, focusing solely on the inertia time constant overlooks critical decision-making information such as reserve capacity and frequency regulation coefficient.

In conventional power sources, the inertia time constant of a generating unit depends only on its physical parameters and can be regarded as an inherent attribute. In renewable energy generation systems, the GFM can adjust its virtual inertia time constant through control strategies, however, such adjustment must be based on sufficient reserve capacity. Since modern power grids incorporate both conventional power sources and a significant share of renewable energy, relying solely on the inertia time constant is no longer sufficient for comprehensively assessing the system’s frequency response capability. The FSM encompasses both the inertia time constant and the dynamic process of FCAS, better aligning with the requirements for frequency stability evaluation in contemporary power systems.

The physical significance of the FSM presented in this paper is reflected in two key aspects. Firstly, regarding RoCoF, the FSM illustrates the relationship between the acceptable power deficit and system inertia. Secondly, with respect to the lowest frequency point, FSM reflects the power grid frequency stability boundary under the combined action of tolerable power deficit, system inertia, and FCAS dynamic regulation. Thus, FSM can incorporate more comprehensive decision-making information. Moreover, as mentioned in the introduction, FSM can directly characterize the tolerable power deficit under frequency constraints, thereby providing an intuitive reference for reserve capacity configuration in grid planning or operational strategies.

**Fig 1:**
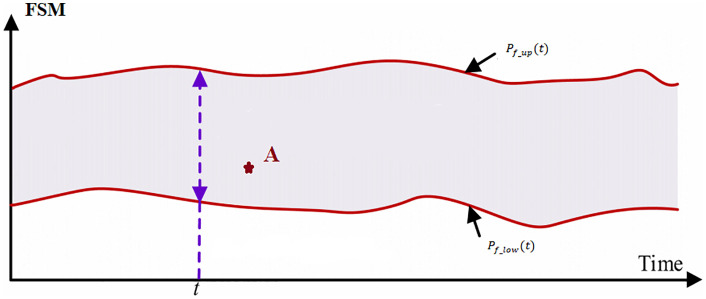
Schematic diagram of the FSM.

FCAS responses are typically completed on a timescale of seconds to minutes, while the 1-hour timeframe targets the unit commitment scheduling cycle. Within this cycle, the system topology and the number of online units are fixed; thus, the relevant parameters can be considered time-invariant, which serves as the foundation for the analysis in this paper. This study focuses on analyzing the FSM during specific periods or instances, rather than examining the maximum or minimum FSM across all timeframes or under extreme boundary conditions. This approach is more practical where operational personnel can formulate strategies for the next time period based on the FSM of the current period, thereby enhancing the security and adaptability of grid operation. As Fig 1 depicts, although the resultant value (Point A) may not represent the global minimum, it nonetheless quantifies the instantaneous FSM magnitude corresponding to the prevailing operational state.

## 3 Factors influencing FSM and critical inertia threshold analysis

Three primary determinants govern the magnitude of FSM: 1) the operational scale of the generation units, 2) the system’s equivalent inertia M_sys_, and 3) imposed constraint conditions. The system frequency dynamics during a disturbance were modelled using the aggregated swing equation [15]:


$$M_{sys}\frac{d\Delta f(t)}{f_{\text{0}}dt}+D\Delta f(t)=\Delta P_{m}(t)-\Delta P_{e}(t)$$ (2)

where Δf(t) represents the frequency deviation, $M_{sys}\;$ represents the equivalent inertial time constant of power systems. D represents the System damping constant. $\Delta P_{m}(t)$ and $\Delta P_{e}(t)$ represent the mechanical power and electromagnetic power, respectively.

### 3.1 The operational scale of the generation unit

Expanding the right-hand side of the swing equation yields the following formulation:


$$\begin{array}{c} P_{f_{up}}(t)=max\left(\sum_{i}^{N_{SG}}{\Delta P}_{SG,i}(t)+\sum_{j}^{N_{CIG}}{\Delta P}_{CIG,j}(t)\right) \end{array}$$ (3)

$$\begin{array}{c} P_{f_{low}}(t)=min\left(\sum_{i}^{N_{SG}}{\Delta P}_{SG,i}(t)+\sum_{j}^{N_{CIG}}{\Delta P}_{CIG,j}(t)\right) \end{array}$$ (4)

where $\Delta P_{SG,i}(t)$ represents the power deficit of SG, ${\Delta P}_{CIG,j}(t)$ represents the power deficit of CIG, N_SG_ represents the total number of online SG, and N_CIG_ represents the total number of online CIG. This equation indicates that the FSM is determined by the online capacity and the control strategies of generation units.

According to the definition of FSM, this index represents a differential value quantifying the reserve margin of power reservation. Consequently, the load side is not considered a source of reserve power, and the FSM primarily reflects the reserve capacity on the generation side. Synchronous generators can promptly compensate for power deficits through inherent inertia and primary frequency regulation. Similarly, the GFM can emulate the power characteristics of synchronous generators to mitigate power shortfalls immediately. In contrast, the GFL typically employs constant power control mode [16], and does not contribute to frequency support. Hence, this paper focuses exclusively on the frequency regulation contribution of GFM within the considered CIG system.

### 3.2 Equivalent inertial time constant

As derived from the left-hand term of the swing equation, the FSM is directly proportional to M_sys_, which aggregates rotational inertia from the SG and emulates inertia from the CIG. An increase in M_sys_ corresponds to higher FSM values and enhanced grid frequency stability. The following section primarily discusses the influence of virtual inertia on the equivalent inertia time constant. The CIG includes both GFM and GFL units.

The GFL mitigates power imbalance in the system by adjusting its output power, thereby reducing the rate of frequency change. However, this mechanism constitutes a power response that does not contribute to system inertia. Therefore, the inertial time constant of a grid-following converter is zero.

The GFM emulates the dynamic behavior of synchronous generator rotors and electromagnetic transients via its control strategy, thus exhibiting external operational characteristics similar to those of synchronous generators. This control paradigm classifies the GFM as a voltage source. Accordingly, GFMs provide virtual inertia, and per regulatory guidelines, their inertial time constant is user-definable, typically ranging from 4 to 12 seconds, with 5 seconds recommended [17].

During the development of power grid infrastructures, the distinct generation source configurations differentially influence grid frequency stability. To systematically evaluate the effectiveness of configurations in enhancing frequency stability, the following premises were established: 1) All GFM units maintain identical equivalent inertia time constants. 2) The aggregate energy storage capacity remains fixed. Thus, varying the GFM storage penetration ratios (K) represents an alternative infrastructure development strategy. The GFM storage penetration ratio K is mathematically defined as


$$K=\frac{S_{GFM}}{S_{GFM}+S_{GFL}}=\frac{S_{GFM}}{S_{CIG}}$$ (5)

where S_CIG_ represents the rated capacity of CIG, and S_GFM_ and S_GFL_ represent the rated capacity of GFM and the rated capacity of GFL, respectively.

Under these conditions, the equivalent inertia time constant for low-inertia power systems incorporating both CIG and SG devices can be formulated as


$$M_{sys}=\frac{\sum_{i=\text{1}}^{n}S_{SGi}M_{i}^{SG}+M_{GFM}S_{CIG}K}{\sum_{i=\text{1}}^{n}S_{SGi}+S_{CIG}K}$$ (6)

Where $M_{i}^{SG}$ represents the equivalent inertial time constant of SG, and $M_{GFM}\;$ represents the equivalent inertial time constant of GFM. It should be noted that equation (6) fails to capture inertial variations during dynamic processes and is therefore only suitable for preliminary assessment at the planning stage.

The GFM can provide virtual inertia support to low-inertia power systems, where the M_GFM_ parameter has a critical influence on M_sys_. Intuitively, this suggests that the increased penetration (K) of GFM storage converters enhances frequency stability.

Contrary to this intuition, the user-configurable nature of M_GFM_ in GFM introduces a critical consideration where, without proper parameter coordination, excessive K values may paradoxically reduce Msys, thereby degrading frequency stability.

Through analytical derivation, we establish that M_sys_ maintains a positive correlation with K only when the M_GFM_ satisfies the following constraint condition: Under this criterion, increasing the GFM storage penetration (K) proportionally enhances M_sys_, thereby preserving the frequency stability.


$$M_{GFM}>\frac{\sum_{i=\text{1}}^{n}S_{SGi}M_{i}^{SG}}{\sum_{i=\text{1}}^{n}S_{SGi}}$$ (7)

The equality condition in equation (7) defines the critical threshold value of M_GFM_ as M_x_.

**Proof:** Building on equation (6), demonstrating that M_sys_ is functionally dependent on K, the formulation can be re-expressed as


$${f(k)=M}_{sys}=\frac{A+M_{GFM}k}{B+k}$$ (8)

where


$$\{A=\sum_{i=\text{1}}^{n}S_{SGi}M_{i}^{SG}\;B=\sum_{i=\text{1}}^{n}S_{SGi}\;k=S_{CIG}K\;$$

To ensure f(k) constitutes a monotonically increasing function, the derivative condition $\frac{df(k)}{dk}>\text{0}$ must be satisfied to ensure that f(k) constitutes a monotonically increasing function


$$\frac{df(k)}{dk}=\frac{BM_{GFM}-A}{{\left(B+k\right)}^{\text{2}}}>\text{0}$$ (9)

This inequality resolves to:


$$M_{GFM}>\frac{A}{B}$$ (10)

Thus, the theoretical derivation presented in equation (7) of the main text was rigorously validated.

### 3.3 Constraint conditions

As demonstrated by equation (1), the constraint conditions represent another critical determinant of FSM magnitude.

Considering that the governor responses are not activated during the first stage of the inertial response, the maximum RoCoF $R_{f}$ is expressed as follows [14]:


$${\frac{d\Delta f}{dt}|}_{max}=R_{f}=-f_{\text{0}}\frac{P_{rocof}}{M_{sys}}$$ (11)

Through algebraic transformation, the equation becomes:


$$P_{rocof}=-\frac{{R_{f}M}_{sys}}{f_{\text{0}}}$$ (12)

Recent revisions to grid codes have proposed relaxing RoCoF protection settings to accommodate the integration of non-synchronous resources better and reduce the risk of grid separation. The UK, Ireland, and Northern Ireland grid operators have implemented 1 Hz/s RoCoF ride-through standards [18,19]. Regarding frequency thresholds, China’s third defense line configuration requires maintaining a minimum frequency of 49.0 Hz during first-level contingencies [20], while Spain specifies 49.2 Hz [21].

For analytical clarity, it is postulate $P_{rocof}>P_{\Delta f}$ in subsequent discussions. Thus, $P_{\Delta f}$ is designated as the index for FSM quantification.

### 3.4 Critical inertia threshold and system planning implications

**Fig 2:**
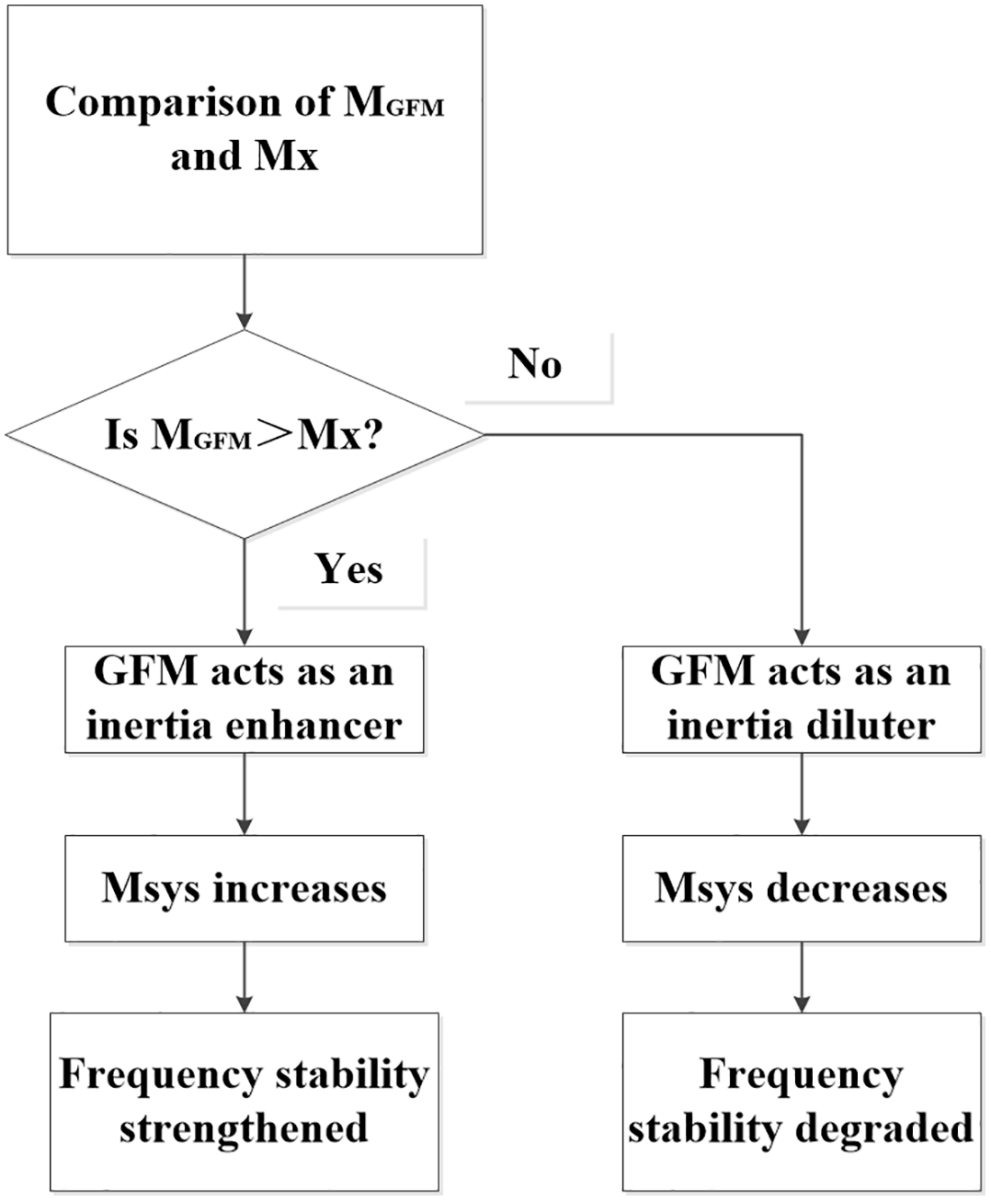
Impact of M_GFM_ on frequency stability.

Equation (7) reveals a non-intuitive but critical insight where increasing the penetration of GFM storage (K) does not necessarily lead to an enhancement in system inertia. The efficacy of GFM integration is contingent upon the relative magnitude of its configured virtual inertia (M_GFM_) in comparison to the critical threshold Mx. Fig 2 illustrates the impact of M_GFM_ on frequency stability.

Physical interpretation: The critical inertia Mx is mathematically defined as the capacity-weighted average inertia of the existing synchronous generator fleet. Physically, it represents the existing system’s “inertial baseline” or “inertial density.”

When M_GFM_ > Mx: Each additional unit of GFM capacity introduces a higher-than-average inertial contribution, consequently, increasing the penetration K raises the overall system inertia (Msys), strengthening frequency stability. In this sense, the GFM acts as an inertia enhancer.

When M_GFM_ < Mx: Each additional unit of GFM capacity introduces a lower-than-average inertial contribution. In this scenario, diluting the grid with such GFM units reduces the overall system inertia (Msys), potentially degrading frequency stability despite increased “green” capacity. Whereby, the GFM inadvertently acts as an inertia diluter.

This finding provides concrete guidance for system planners and operators. For grid planners, planning new GFM storage projects should begin by calculating Mx based on the existing or expected future synchronous-generator fleet. The virtual inertia constant M_GFM_ for new GFMs should then be specified to be significantly greater than this calculated Mx to ensure they provide genuine inertia support. This may influence inverter sizing and the selection of control strategies. For Grid Operators, Mx should be treated as a dynamic quantity that changes with the set of committed synchronous units. Therefore, the requirement M_GFM_ > Mx should be evaluated dynamically. Operating strategies must ensure that the aggregate virtual inertia from online GFMs consistently contributes positively to Msys.

## 4 Evaluation of the FSM

According to the analysis in Section 3, multiple factors affect the magnitude of FSM values. This section examines the methodology for calculating the FSM utilizing both aggregation models and PMU.

### 4.1 FSM evaluation based on the aggregation model

According to the preceding context, the GFL cannot provide inertia support. Consequently, during power disturbances, inertia in low-inertia power systems is supplied by a synchronous generator (SG) and the GFM.

1) SG: A transfer function for the frequency-power relationship of SG is obtained from [22] as (13).


$$\left(M_{i}^{SG}s+D_{i}^{SG}\right)\Delta f_{i}=-\Delta P_{i}+{\Delta P}_{Gi}^{SG}$$ (13)

Where


$${\Delta P}_{Gi}^{SG}=\frac{-K_{i}^{SG}}{\text{1}+sT_{i}^{SG}}\Delta f_{i}$$ (14)

Where$\;\Delta P_{i}$ represents the difference between mechanical and electrical power,$\;{\Delta P}_{Gi}^{SG}$ represents the output of SG, $D_{i}^{SG}$ represents the damping contribution from SG and frequency-dependent loads, $K_{i}^{SG}$ represents the inverse of the droop of SG, $T_{i}^{SG}$ represents the time constant of the governor.

Following a step disturbance, governor systems and prime movers are typically represented as sustaining constant output power, while the effects of load damping are often disregarded. Under these assumptions, the frequency deviation is derived from the standard equation (15) as indicated by Baldwin and Schenkel [23].


$$\frac{d\Delta f_{i}}{dt}\approx \frac{-\Delta P_{i}}{M_{i}^{SG}}=-m^{SG}$$ (15)

Where ${\;m}^{SG}$ represents the inverted sign frequency derivative of SG.

Let us assume the additional simplification that the output given by (15) could be approximated by an averaged constant ramp:


$${\Delta P}_{Gi}^{SG}(t)=G_{i}^{SG}\cdot m^{SG}\cdot t$$ (16)

Where $\;G_{i}^{SG}$ represents the ramp gain of SG. Substituting equation (14) into equation (13) yields the following formula:


$${\Delta P}_{Gi}^{SG}(t)=K_{i}^{SG}\cdot m^{SG}\cdot (t-{T_{i}}^{SG}+{T_{i}}^{SG}e^{-t/{T_{i}}^{SG}})$$ (17)

Where


$$G_{i}^{SG}=K_{i}^{SG}(\text{1}-\frac{{T_{i}}^{SG}}{t_{p}}(\text{1}-e^{\frac{-t_{p}}{{T_{i}}^{SG}}}))$$ (18)

2) GFM: The GFM droop and its LPF for the measured output power are combined to derive a swing (19) for GFM inverters [24].


$$\left(M_{GFM}s+D_{i}^{GFM}\right)\Delta f_{i}=-\Delta P_{i}+{\Delta P}_{Gi}^{GFM}$$ (19)

It is known that there is always a significant delay in the mechanical governor of SG. In previous research on VSG, this delay is also imitated when (20) is applied [25–27].


$${\Delta P}_{Gi}^{GFM}=\frac{-K_{i}^{GFM}}{\text{1}+sT_{i}^{GFM}}\Delta f_{i}$$ (20)

Assuming a further simplification, the output described in equation (20) may be approximated as the constant averaged ramp presented in (21).


$${\Delta P}_{Gi}^{GFM}(t)=G_{i}^{GFM}\cdot m^{GFM}\cdot t$$ (21)

Where $G_{i}^{GFM}$ and $G_{i}^{SG}$, $m^{GFM}$ and $m^{SG}$ have similar expressions and definitions.

3) The Aggregation Model: The SG and GFM models are aggregated to form a comprehensive model (22) for low-inertia power systems, where the frequency transitions to the COI frequency while neglecting local frequency oscillations around the COI.


$$\left(M_{sys}s+D_{sys}\right)\Delta f_{COI}=-\Delta P_{T}^{SG+GFM}+{\Delta P}_{G}^{SG+GFM}$$ (22)

When the damping of the loads is neglected. Equations (23) and (15) have similar expressions.


$$\frac{d\Delta f_{COI}}{dt}\approx \frac{-\Delta P_{T}^{SG+GFM}}{M_{sys}}=-m$$ (23)

Where$\;\Delta P_{T}^{SG+GFM}$ represents the difference between mechanical and electrical power, $\;{\Delta P}_{G}^{SG+GFM}$ represents the output of SG and GFM, $D_{sys}$ represents the damping of power systems, $K_{i}$ represents the inverse of the droop of power systems, $T_{i}$ represents the time constant of power systems, m represents the inverted sign frequency derivative of power systems, $G_{i}$ represents the ramp gain of power systems. Equation (22) becomes


$$M_{sys}\frac{d\Delta f_{COI}}{dt}=-\Delta P_{T}^{SG+GFM}+\sum_{i}^{N_{SG}+N_{GFM}}G_{i}\cdot m\cdot t$$ (24)

where


$$G_{i}=K_{i}(\text{1}-\frac{T_{i}}{t_{p}}(\text{1}-e^{\frac{-t_{p}}{T_{i}}}))$$ (25)

Solving (24) in time yields


$$\Delta f_{COI}(t)=-\frac{\Delta P_{T}^{SG+GFM}}{M_{sys}}\cdot t+\frac{\text{1}}{{\text{2}M}_{sys}}\sum_{i}^{N_{SG}+N_{GFM}}G_{i}\cdot m\cdot t^{\text{2}}$$ (26)

Minimum is found by solving the following:


$$\frac{d\Delta f_{COI}}{dt}=\text{0}$$ (27)

$\;The\;nadir\;time\;t_{p}\;$ is obtained:


$$t_{p}=\frac{M_{sys}}{\sum_{i}^{N_{SG}+N_{GFM}}G_{i}}$$ (28)

Substituting (28) in (26), the maximum frequency deviation $\Delta f_{m}\;$ is found:


$$\Delta f_{m}=\frac{-\Delta P_{T}^{SG+GFM}}{\text{2}\sum_{i}^{N_{SG}+N_{GFM}}G_{i}}\;\;(p.u.)$$ (29)

When the maximum frequency deviation reached its permissible limit, the computational formula for $P_{\Delta f}$ was derived as follows:


$$P_{\Delta f}=-\Delta f_{m}\cdot \text{2}\cdot \sum_{i}^{N_{SG}+N_{GFM}}G_{i}$$ (30)

### 4.2 FSM evaluation based on PMU

Given the fixed number of committed units and constant unit parameters during a specific time period, G_i_ becomes a fixed constant according to Equation (31). Therefore, the FSM for this period can be simplified to:


$$P_{\Delta f}=-\Delta f_{m}\times K_{f}$$ (31)

Where $K_{f}$ represents the frequency proportionality coefficient.

This formulation provides the theoretical basis for the preliminary assessment of the FSM. Specifically, when a power disturbance occurs at a particular node under given system conditions, provided that the PMU-recorded threshold values have been reached, the FSM can be estimated using disturbance power and frequency deviation data derived from PMU measurements under identical nodal operating conditions.

## 5 Simplified assumptions and error analysis

### 5.1 Simplified assumptions

As described in Section 4.1, the FSM calculation method based on the aggregate model (Equation 30) relies on several simplified assumptions. First, the frequency regulation effect of loads is neglected, and the system damping constant is assumed to be zero during the derivation. Second, the dynamic responses of power sources are aggregated and linearized, meaning that the complex and heterogeneous frequency response behaviors of SGs and GFMs are approximated by a uniform linear response with a constant ramp rate Gi. Third, the method adopts the center-of-inertia (COI) frequency consistency assumption, where the system frequency is considered to follow the COI frequency strictly while local frequency oscillations induced by disturbance locations and network structure are ignored.

These simplifications significantly reduce model complexity and computational burden, making the method suitable for rapid scanning in planning stages and preliminary assessments in online applications. However, in modern low-inertia systems, the dynamic characteristics of loads and the diverse control strategies and response speeds of various power sources make the errors introduced by these assumptions non-negligible.

### 5.2 Applicability boundaries and error analysis

The analytical framework developed in this study relies on several simplifying assumptions that enable tractable estimation of frequency stability index. While these assumptions are widely used in reduced-order frequency response models, their validity depends on specific system conditions and may introduce non-negligible errors when those conditions are not satisfied. To clarify the scope and limitations of the proposed approach, this section outlines the applicability boundaries of the key assumptions and identifies the primary sources of error associated with each. The discussion provides guidance for interpreting the results, especially in low-inertia or heterogeneous power systems where model deviations can significantly affect FSM estimates.

1) Load Damping Effect

Assumption: During the initial stage of inertial response, the load power is assumed to be independent of frequency changes (i.e., D = 0).

Applicability Boundary: This assumption is held in scenarios where motor loads constitute a relatively small proportion.

Error Analysis: A higher proportion of motor loads increases the error of this assumption. The self-regulating effect of motor loads provides positive damping power, which helps suppress frequency decline. Ignoring this effect leads to an underestimation of the nadir frequency and consequently an overestimation of the tolerable power deficit for maintaining frequency within limits, thereby overestimating the FSM.

2) Linear Aggregation of Power Source Responses

Assumption: The inertial responses of SGs (governor and prime mover systems) and GFMs (virtual governor systems) with different time constants are aggregated into a single equivalent linear ramp response.

Applicability Boundary: This assumption is valid when the system is dominated by similar types of power sources with comparable time constants.

Error Analysis: Greater diversity in power source types and control parameters increases the error. GFMs exhibit virtual inertial responses typically within milliseconds, whereas SGs have governor response delays ranging from hundreds of milliseconds to several seconds. The linear aggregation model fails to accurately capture these temporal differences in response, resulting in deviations in predicting the nadir frequency and, consequently, errors in FSM estimation.

3) COI Frequency Consistency Assumption

Assumption: The system frequency is assumed to be uniform, ignoring the effects of network topology and disturbance location.

Applicability Boundary: This assumption applies to systems with strong connectivity and relatively small-scale disturbances.

Error Analysis: In low-inertia systems, reduced overall stiffness results in significant variations in the RoCoF and frequency deviation across different locations for the same disturbance. When evaluating disturbances occurring at electrically remote or weak nodes, the aggregate COI-based model severely underestimates the local RoCoF and frequency deviation, thereby overestimating the FSM for that specific disturbance location.

## 6 Application of the FSM

In this section, the application of the proposed FSM is discussed. The frequency stability of the system was analyzed by comparing the lower boundary of the FSM with the maximum disturbance power of the system.

The application framework of the FSM based on the aggregation model is described in Section 6.1, while the application framework of the FSM based on PMU is described in Section 6.2. In Section 6.3, the advantages and disadvantages of these two algorithms are compared and analyzed.

### 6.1 Application framework of the FSM based on aggregation model

To operationalize the proposed FSM assessment method, a structured evaluation procedure is employed. The procedure integrates real-time system information, analytical formulations derived earlier, and a stability verification criterion to determine whether the system can withstand a specified disturbance. The following steps summarize the implementation workflow, from parameter initialization to FSM computation and final stability judgement.

Step 1: System Parameter Initialization

The system parameters are initialized first, including setting the $R_{f}$ value according to the given standard, obtaining the commitment plans for online SG and GFM, calculating the system parameter M_sys_ using PMU, and acquiring relevant parameters G_i_ of these online units.

Step 2: FSM Calculation

The initialized parameters are substituted into equations (30) and (12) to derive the frequency deviation power component $P_{\Delta f}$ and RoCoF power component $P_{rocof}$. Compute $P_{f}$ using equation (1).

Step 3: Stability Criterion Validation

**Fig 3:**
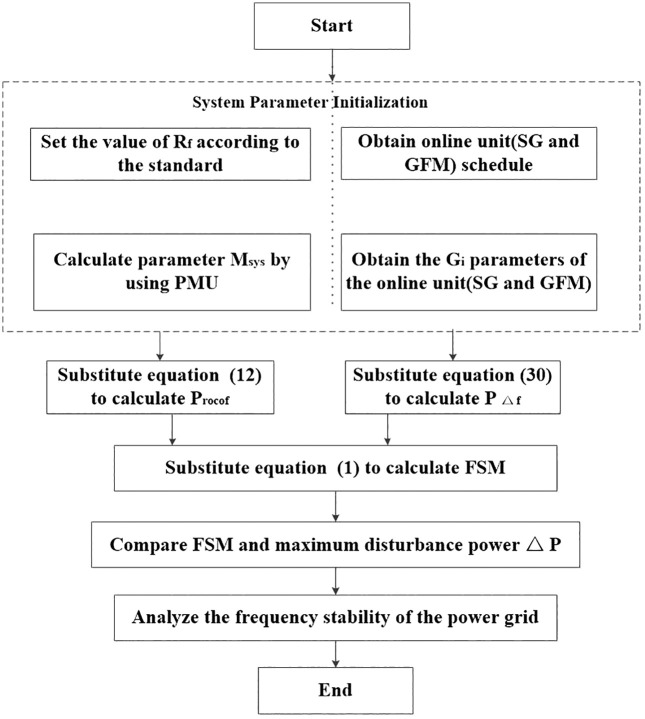
Application framework of the FSM based on aggregation Model.

Comparison of $P_{f}$ with maximum disturbance power ΔP in the operational system. If $P_{f}$ ≥ ΔP, the system frequency is deemed stable; otherwise, the frequency control strategies or inertia enhancement measures are activated. Fig 3 presents the application framework of the FSM based on the aggregation model.

### 6.2 Application framework of the FSM based on PMU

It should be noted that the FSM derived from practical algorithms represents an instantaneous value under specific operating conditions, rather than a conservative lower bound. The operational workflow aligns with the aggregated model approach in three sequential phases: system parameter initialization, FSM computation, and validation of the stability criterion. Although the latter two phases remain identical, the parameter initialization diverges significantly.

The framework is initiated with minor disturbances by employing a dual-path parallel processing mechanism for dynamic parameter coupling:

Left Branch: Real-time PMU data drives the transient parameter M _sys_ calculation, enabling RoCoF power component $P_{rocof}$ derivation via equation (12).

Right Branch: Sampled disturbance power ΔP and frequency deviation Δf facilitate iterative system damping coefficient K_f_ computation through equation (31). Subsequently, substituting the rated frequency deviation Δf_m_ into equation (31) yields the frequency deviation power component $P_{\Delta f}$.

**Fig 4:**
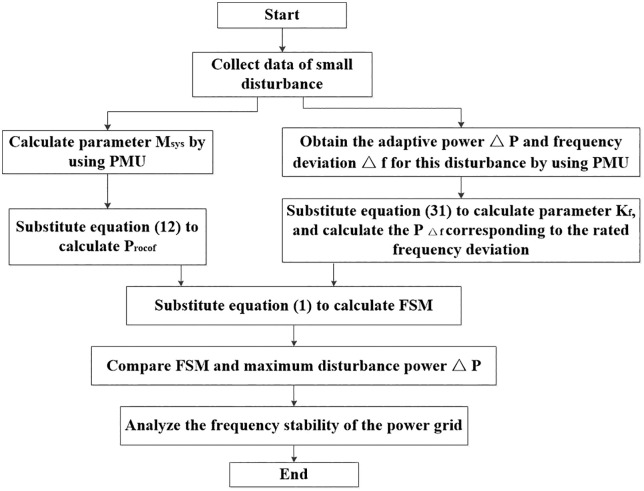
Application framework of the FSM based on PMU.

This bifurcated initialization enables concurrent parameter estimation. Fig 4 presents the application framework of the FSM based on PMU.

The frequency proportionality coefficient $K_{f}$ can be calculated using an iterative algorithm based on the gradient descent method. The specific computational procedure is as follows:

**Step 1: Initialize Parameters.** Set the initial value for the frequency proportionality coefficient $K_{f\text{0}}$, the convergence threshold ∊, the maximum number of iterations N_max_, and the learning rate α.

**Step 2: Preprocess PMU Data.** Filter the raw PMU measurements (e.g., using a low-pass filter) to suppress high-frequency noise and extract the data segment surrounding the power disturbance event for subsequent calculations.

**Step 3: Iterative Calculation Core Loop.**

**Compute Error Function** E_k_: Calculate the least squares error based on the current estimate $K_{fk}$and the preprocessed PMU data (ΔP_i_, Δf_i_).

**Update Estimate**: Using the gradient descent method, compute the gradient of the error function ∂K_f_∂E_k_ and update the damping coefficient estimate K_fk+1_ = K_fk_ − α ⋅ ∂K_f_∂E_k_.

**Convergence Check**: Check if the update in this iteration is less than the preset convergence threshold ∊.

If **Yes**, the algorithm is considered converged. Exit the loop and output the final $K_{f}$.

If **No**, check if the maximum iteration count N_max_ has been reached.

If **Yes**, terminate the iteration to prevent an infinite loop and output the current result (a warning can be issued).

If **No**, return to step 3 to continue with the next iteration.

**Fig 5:**
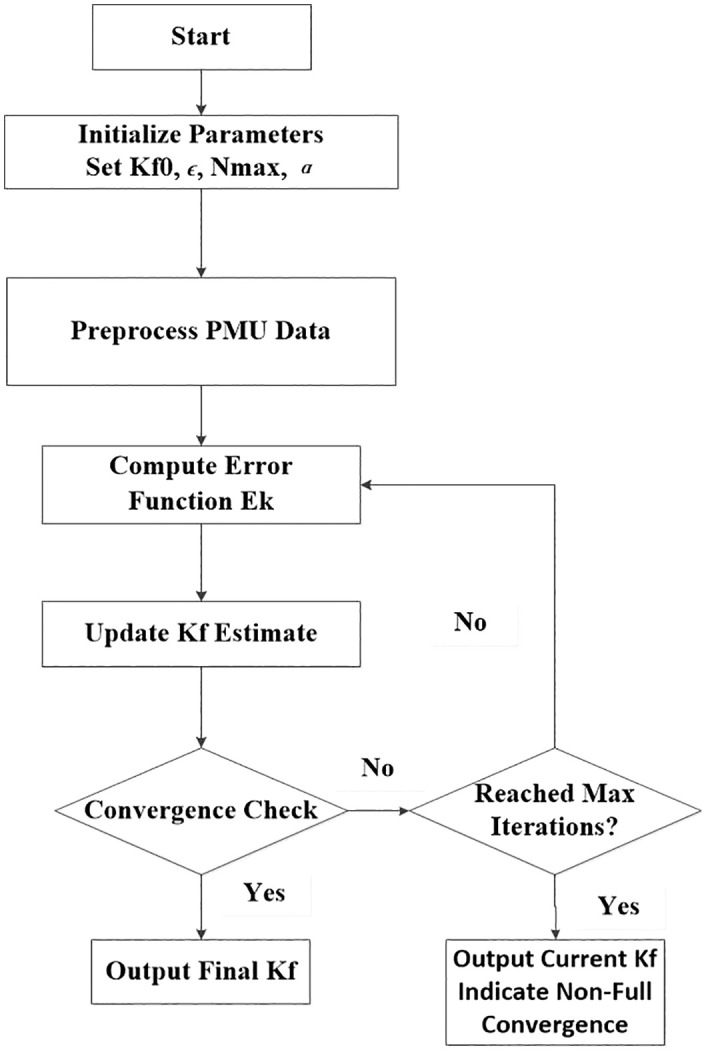
An iterative algorithm for the frequency proportionality coefficient.

Since the error function is convex, the algorithm guarantees convergence to the global minimum, typically achieved within several tens of iterations. A data preprocessing step (low-pass filtering) is incorporated to effectively suppress high-frequency noise, thereby reducing its impact on the iterative process. The gradient descent method further enhances robustness by averaging out the effects of noise over multiple iterations. Through data preprocessing and appropriate parameter selection, the algorithm can be effectively applied to practical power system FSM assessments. Fig 5 presents the iterative algorithm of the frequency proportionality coefficient.

### 6.3 Comparison of advantages and disadvantages between the two calculation methods

The two FSM calculation methods proposed in this paper are not mutually exclusive; instead, they are complementary to each other. Table 1 quantifies the comparison between the two calculation methods. Together, they form a full-cycle frequency stability assessment system from planning to operation:

**Table 1:** Comparison Between the Two Calculation Methods.

Comparison Dimension	Aggregation Model-Based Method	PMU-Based Method
**Applicable Stage**	Planning stage, offline analysis, strategy formulation	Real-time operation, online monitoring, rapid assessment
**Data Requirements**	System parameters, unit models, droop coefficients, etc.	PMU measurement data, actual disturbance records
**Calculation Accuracy**	High (theoretically sound, but relies on parameter accuracy)	Medium (affected by disturbance scale; larger errors under minor disturbances)
**Real-Time Performance**	Low (complex computation, unsuitable for high-frequency updates)	High (can rapidly respond to system changes)
**Dependence on Disturbance**	Independent of actual disturbances	Must rely on actual disturbance events
**Output Results**	Theoretical FSM lower limit (conservative value)	Instantaneous FSM (estimated value at the current operating point)

**Planning Stage:** The aggregation model-based method should be employed to perform FSM calculations for multiple scenarios and parameters. This is used to assess the impact of renewable energy penetration, GFM inertia configuration, reserve capacity, and other factors on frequency stability.

**Operation Stage:** The PMU-based method should be integrated, utilizing actual disturbance data to update the FSM estimate continuously. This provides a basis for real-time adjustments to unit commitment and optimization of GFM control strategies.

Ultimately, through the mode of “offline calculation + online calibration,” the FSM can effectively serve the safe and stable operation of low-inertia power systems.

## 7 Case studies

In this section, two test systems are used to validate the effectiveness of the proposed FSM: a realistic provincial power grid in China, and a modified IEEE 39-bus system. All experiments were performed on a personal computer with an Intel I Core I i7-7500U (2.9 GHz) processor and 16 GB of memory, using Power Factory/Matlab-Simulink.

For analytical convenience, the simulation framework assumed that the RoCoF parameter exceeded the $P_{\Delta f}$ value. Consequently, $P_{\Delta f}$ serves as the FSM throughout the subsequent evaluations.

### 7.1 Provincial-level power grid planning

**Fig 6:**
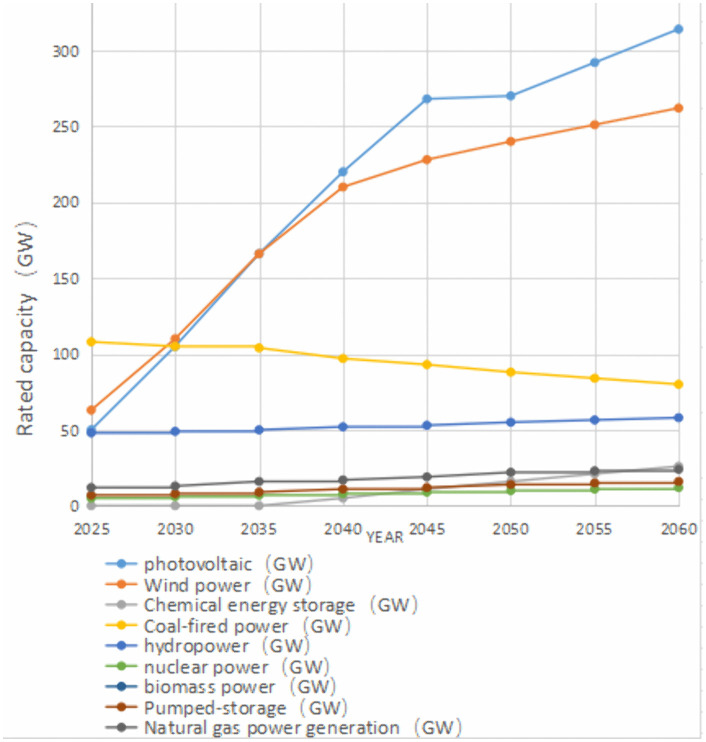
Installed capacity of various power sources.

Fig 6 presents the projected installed capacity mix (2025–2060) for a Chinese provincial grid with a maximum DC block capacity of 8 GW. The plan provides the online unit commitment schedule, along with the operating parameters and control strategies for each unit. The rated frequency deviation Δf_m_ = −1 Hz (per unit: −0.02).

**Fig 7:**
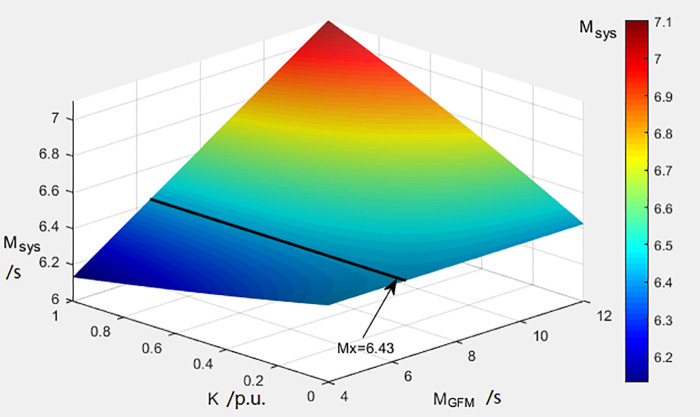
Changes in the inertia time constant of the power system.

Matlab-Simulink simulations for the 2060 carbon neutrality scenario establish M_x_ = 6.43 s, as per regulatory guidelines and equation (7). Fig 7 illustrates the M_sys_ variations across the GFM storage penetration ratios (K: 0–1) and the GFM inertia time constants (M_GFM_: 4–12 s).

Key observations:

For M_GFM_ < M_x_, M_sys_ decreases with rising K values.

For M_GFM_ > M_x_, M_sys_ increases proportionally with K, demonstrating enhanced frequency stability with higher grid-forming storage penetration.

Table 2 quantifies the FSM variations under different M_GFM_ and K combinations. Both M_GFM_ = 8 s and 12 s satisfy the critical threshold requirements, with the FSM increasing alongside the M_GFM_ and K values. According to Table 2, an increase in the M_GFM_ correlates with a rise in the FSM. With a fixed M_GFM_ value, increasing K results in a higher FSM, indicating improved frequency stability in the power grid.

**Table 2:** FSM values under different parameters.

K (p.u.)	M_GFM_ (s)	M_sys_ (s)	FSM (GW)	t_p_ (s)
0	8	6.43	11.47	8.77
0.5	8	6.53	12.28	8.90
1	8	6.62	13.07	9.00
0	12	6.43	11.47	8.77
0.5	12	6.79	12.29	9.21
1	12	7.10	13.12	9.59

**Fig 8:**
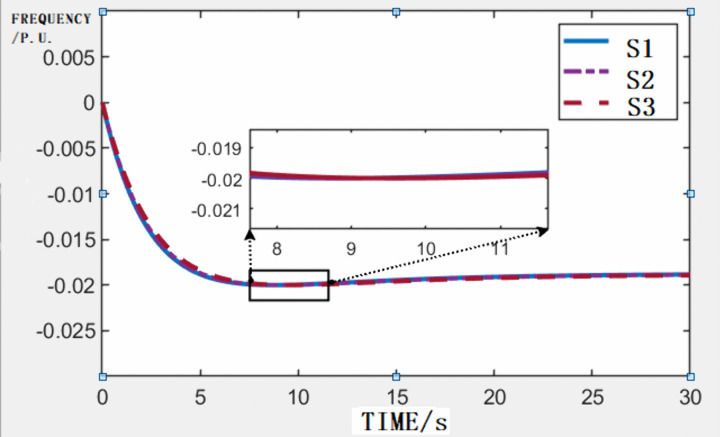
Frequency changing curve.

Three representative scenarios are simulated (Fig 8):

S1: K = 0, M_GFM_ = 8 s.

S2: K = 1, M_GFM_ = 8 s.

S3: K = 1, M_GFM_ = 12 s.

A simulated 0 s power deficit drives the system frequency to the lower limit (−0.02 p.u.). Comparative FSM analysis reveals that the minimum frequency stability margin (11.47 GW) exceeds the maximum DC block disturbance (8 GW), thereby validating the grid’s resilience under critical contingency scenarios. These results demonstrate compliance with operational safety standards and confirm the viability of the proposed capacity expansion strategy.

### 7.2 IEEE 39-Bus system

**Fig 9:**
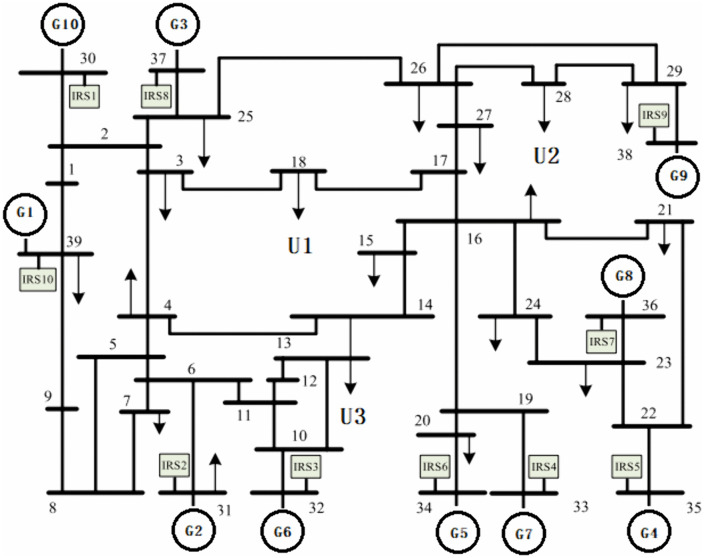
Diagram of the modified IEEE 39-bus system.

Fig 9 illustrates the modified IEEE 39-bus system [28] with a nominal frequency of 50 Hz. Key modifications to the original system include the following.

1) Adjusted load distribution and synchronous generator parameters;
2) Integration of five GFM energy storage units (IRS1-IRS5);
3) Addition of five GFL energy storage units (IRS6-IRS10).

The generator specifications are summarized in Table 3, which includes conventional generators employing IEEE G1-type speed governor systems, where a more detailed control architecture can be found in Peng et al. [29]. The virtual synchronous generator (VSG) control strategy for energy storage units follows the methodology outlined in Zhong [30]. RoCoF and rated frequency deviation are configured at 0.5 Hz/s and 0.5 Hz, respectively, with a data sampling rate of 100 samples per second. FSM_agg denotes the FSM value derived from the aggregation-based model, while FSM_sim represents the FSM value obtained from the PSS/E simulation model [31]. The disturbance location U1 is the farthest from the PMU installation site, U2 is at an intermediate distance, and U3 is the closest.

**Table 3:** Generator parameters of the modified IEEE 39-bus system.

Number	Rated capacity (MW)	Inertia constant (s)
G01	1200	5
G02	700	4.329
G03	800	5
G04	800	5
G05	300	4.333
G06	800	4.35
G07	700	3.711
G08	700	5
G09	1000	3.45
G10	1000	4.2

Three operational scenarios are established to evaluate the FSM under varying conditions:

S1: Total grid capacity = 10000 MW, K = 0%, M_GFM_ = 0 s and M_sys_ = 4.44 s.

S2: Expanded grid capacity = 16000 MW with increased renewable penetration, K = 50%, M_GFM_ = 3 s and M_sys_ = 3.96 s.

S3: Identical parameters to S2, except M_GFM_ = 5 s and M_sys_ = 4.63 s.

The scenario-specific parameters are systematically listed in Table 4. This configuration enables a comparative analysis of FSM variations across distinct stages of renewable integration and control parameter configurations.

**Table 4:** Key parameters of different scenarios.

Scenario	K (%)	Rated capacity of SG (MW)	Rated capacity of CIG (MW)	M_GFM_ (s)	M_sys_ (s)	M_sys_ (MW·s)	FSM_sim (MW)
S1	0	8000	2000	0	4.44	44400	300
S2	50	8000	8000	3	3.96	63360	395
S3	50	8000	8000	5	4.63	74808	415

As shown in Table 4, the key indices for the three scenarios includes M_sys_ (s), M_sys_ (MW·s), and FSM_agg. Comparing scenarios S1 and S2, it is observed that M_sys_ (s) exhibits a decreasing trend. Without coupling this index with system capacity, one might mistakenly conclude that the system’s frequency stability is reduced. This highlights a limitation of M_sys_ (s), where, in the absence of system capacity information, it becomes impossible to accurately assess frequency stability. According to Table 4, M_sys_ (MW·s) exhibits an increasing trend, indicating an improvement in the system frequency stability. However, this index still fails to provide grid operators with intuitive decision-making information. Moreover, M_sys_ solely reflects system inertia and does not incorporate critical decision-related data, such as frequency regulation power. Therefore, the FSM proposed in this study offers a more comprehensive representation of frequency stability and delivers intuitively actionable information regarding capacity deficit to grid operators. In other words, the FSM value represents the capacity limit beyond which system frequency instability may occur due to generation tripping or load variation. Grid operators must accordingly adjust the reserve capacity of conventional units, energy storage systems, or the control strategies of GFM inverters to maintain stability.

Comparative analysis of scenarios yields critical insights:

S1 vs. S2: Despite the lower inertia time constants in S2 owing to increased renewable penetration (50% vs. 10% GFM capacity ratio), the FSM values show enhanced stability (Table 4). This counterintuitive result underscores the effectiveness of renewable-integrated frequency support strategies and storage responses in maintaining grid stability.

S2 vs. S3: S3 achieves further FSM improvement thanks to properly configured GFM inertia constants (5s vs the critical threshold). Conversely, S2’s subcritical inertia setting (3s) causes FSM degradation despite identical renewable penetration levels, underscoring the need for optimized inertia time constants to preserve stability.

These findings validate the efficacy of the FSM in low-inertia power system analysis and emphasize the critical relationship between grid-forming converter parameter tuning and system-wide stability enhancement. The methodology successfully bridged the theoretical modeling and practical implementation requirements of modern power systems.

To verify the proposed simplified FSM estimation approach, three load conditions (light, medium, and heavy loads) were subjected to disturbance tests with varying magnitudes: minor (10% fluctuation), moderate (20%), and major (40%). All disturbances were constrained within the frequency thresholds to assess the accuracy of FSM estimation across varying perturbation scales. Following the methodology in Section 6.2, Table 5 presents the quantitative FSM results for each scenario.

**Table 5:** Evaluation of the FSM in different scenarios.

Scenario	Note	FSM based on small disturbance (MW)	FSM based on middle disturbance (MW)	FSM based on big disturbance (MW)	FSM_sim(MW)
S1	U1	328	322	311	300
	FSMerr	9.3%	7.3%	3.7%	/
	U2	325	319	308	300
	FSMerr	8.3%	6.3%	2.7%	/
	U3	317	313	303	300
	FSMerr	5.7%	4.3%	1%	/
S2	U1	431	423	409	395
	FSMerr	9.1%	7.1%	3.5%	/
	U2	427	419	404	395
	FSMerr	8%	6%	2.4%	/
	U3	416	411	398	395
	FSMerr	5.4%	4%	0.8%	/
S3	U1	453	445	430	415
	FSMerr	9.2%	7.2%	3.6%	/
	U2	449	441	425	415
	FSMerr	8.2%	6.2%	2.5%	/
	U3	438	432	420	415
	FSMerr	5.6%	4.1%	1.1%	/

The definitions [32] of the relative error percentage are given by


$${FSM}_{err}(\%)=\left|\frac{{FSM}_{c}-{FSM}_{r}}{{FSM}_{r}}\right|\times \text{1}\text{0}\text{0}\%$$ (32)

$$F_{err}(\%)=\left|\frac{f_{nar,c}-f_{nar,r}}{f_{nar,r}}\right|\times \text{1}\text{0}\text{0}\%$$ (33)

Where ${FSM}_{c}$ represents the calculation value of FSM, ${FSM}_{r}$ represents the reference value of FSM,$\;\;f_{nar,c}\;$represents the calculation value of the nadir frequency, and $f_{nar,r}\;$represents the reference value of the nadir frequency.

For Scenario S1, U3 node exhibited FSM estimation errors of 5.7% (minor disturbance), 4.3% (moderate disturbance), and 1% (major disturbance), demonstrating improved accuracy with increasing disturbance magnitude. All estimations maintained an error of <10%, meeting the engineering tolerance requirements.

The installation location of PMU has a decisive influence on measurement error. Nodes closer to disturbance sources and with stronger signal intensity generally exhibit smaller errors in FSM systems. In contrast, nodes located further from the disturbance sources experience significantly amplified errors, primarily due to the attenuation of disturbances over increased electrical distance. This highlights the need to integrate data from multiple PMUs to improve the overall accuracy of FSM-based measurements.

**Fig 10:**
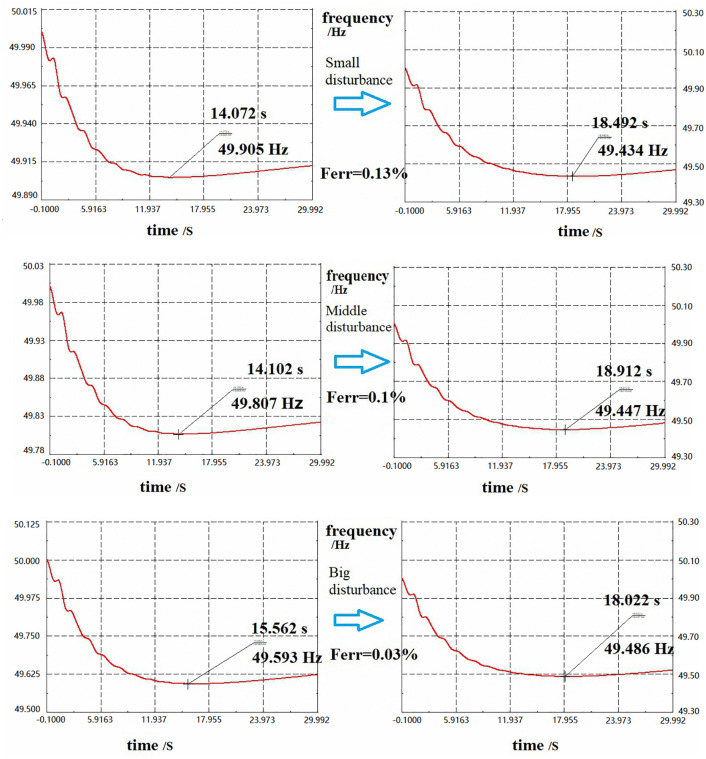
Time domain simulation results of node U1 in scenario S1.

The time-domain simulations for Scenario S1 (U1 node) in Fig 10 reveal the frequency nadir threshold (49.5 Hz) prediction discrepancies via the PMU-Based method, with frequency error remaining below 1%. As can be observed, even when the FSM exhibits a relatively large error (less than 10%), the resulting frequency error remains very small (less than 1%) and occurs only near the boundaries. Therefore, it does not lead to severe frequency instability in the power system.

As shown in Table 6, the aggregated model systematically overestimates the system FSM, with errors within 5%. This phenomenon can be attributed primarily to the insufficient attention given to load damping and the effects of local frequency variations, resulting in an inadequate assessment of the severity of frequency fluctuations.

**Table 6:** The errors of FSM_agg and FSM_SIM.

Scenario	Note	FSM_agg	FSM_sim	FSMerr
S3	U1	430 MW	415 MW	**3.6%**
S3	U2	426 MW	415 MW	**2.7%**
S3	U3	421 MW	415 MW	**1.5%**
S3,50% Motor Load.	U3	440 MW	420 MW	**4.8%**

Load characteristics represent one of the main sources of error. When the dynamics of motor loads are considered in detail, the error reaches 4.8%.

The location of the disturbance has a decisive influence on the error. For nodes close to the disturbance source with strong coupling, the error under the COI assumption is smaller. In contrast, for weak nodes at the grid periphery, the error increases significantly. This indicates that the aggregated model evaluates the “average” performance of the system and may fail to identify the most vulnerable nodes in terms of local stability.

Despite these errors, the aggregated model performs well in trend analysis and maintains high accuracy (error < 5%) within its applicable boundaries.

## 8 Conclusion

The proposed FSM index demonstrates core theoretical value by successfully unifying three critical dimensions that affect frequency stability—system inertia, primary frequency regulation dynamics, and operational constraints—within a concise, quantitative framework. Compared to conventional indices that focus solely on inertial time constants, the FSM advances the field by directly addressing a fundamental question in grid planning and operation, namely, determining the maximum power deficit the system can withstand without losing stability under current conditions. This allows the FSM to move beyond trend-based assessments and provides a physically meaningful boundary value directly applicable to decision-making.

In practical applications, the dual computation framework of the FSM—based on both aggregated modeling and PMU measurements—forms a closed loop from “offline planning” to “online assessment.” The aggregated model approach is suitable for rapid scenario scanning and strategy optimization during the planning stage, while the PMU-based method enables online calibration and real-time evaluation using actual disturbance data during operation. This framework provides grid operators with an unprecedented tool to not only assess the present security level but also predict the system’s disturbance resilience over a forthcoming period (e.g., one hour), thereby facilitating a shift from passive response to active defense.

Parametric analysis reveals critical insights: grid-forming converters require properly calibrated inertia time constants to ensure a positive correlation between grid-forming energy storage penetration and frequency stability improvement, ultimately enhancing system stability.

Numerical studies on both the provincial-level power grid planning and modified IEEE 39-bus system demonstrated the effectiveness of the FSM in frequency stability analysis and its operational value for engineering applications. The proposed index successfully identifies stability vulnerabilities across diverse grid configurations, informs renewable capacity planning, and optimizes protection system settings to ensure secure grid operation.

## Supporting information

S1 File Supporting information file. (DOCX)

## References

[1] Liu L, Luo X, Xiong L, Guo J, Liu X, Liu Y, et al. Preset Power Based Droop Control for Improving Primary Frequency Regulation of Inverters Under Large Disturbances. IEEE Trans Power Electron. 2025;40(7):9153–66. doi: 10.1109/tpel.2025.3547018

[2] Zhang M, Sun L. PLL and Additional Frequency Control Constituting an Adaptive Synchronization Mechanism for VSCs. IEEE Trans Power Syst. 2020;35(6):4920–3. doi: 10.1109/tpwrs.2020.3020725

[3] Duckwitz D, Fischer B. Modeling and Design of $df/dt$ -Based Inertia Control for Power Converters. IEEE J Emerg Sel Topics Power Electron. 2017;5(4):1553–64. doi: 10.1109/jestpe.2017.2703814

[4] Tuttelberg K, Kilter J, Wilson D, Uhlen K. Estimation of Power System Inertia From Ambient Wide Area Measurements. IEEE Trans Power Syst. 2018;33(6):7249–57. doi: 10.1109/tpwrs.2018.2843381

[5] Zhang W, Wen Y, Chung CY. Impedance-Based Online Estimation of Nodal Inertia and Primary Frequency Regulation Capability. IEEE Trans Power Syst. 2023;38(3):2748–60. doi: 10.1109/tpwrs.2022.3186525

[6] Guo J, Wang X, Ooi B-T. Estimation of Inertia for Synchronous and Non-Synchronous Generators Based on Ambient Measurements. IEEE Trans Power Syst. 2022;37(5):3747–57. doi: 10.1109/tpwrs.2021.3134818

[7] Gorbunov A, Dymarsky A, Bialek J. Estimation of Parameters of a Dynamic Generator Model From Modal PMU Measurements. IEEE Trans Power Syst. 2020;35(1):53–62. doi: 10.1109/tpwrs.2019.2925127

[8] Zhai B, Li X, Yang Q, Li W, Sun R, Gao B. Penetration rate-equivalent inertia time constant function identification method of renewable energy power system. In: 2021 IEEE Sustainable Power and Energy Conference (iSPEC). 2021. p. 1–6. doi: 10.1109/ispec53008.2021.9770420

[9] Sun H, Wang B, Li W, et al. Research on inertia system of frequency response for power system with high penetration electronics. Proc CSEE. 2020;40(16):5179–91. doi: 10.13334/j.0258-8013.pcsee.200493

[10] AEMO. Notice of South Australia inertia requirements and shortfall. 2020. [Online]. Available from: https://aemo.com.au/-/media/files/electricity/nem/security_and_reliability/system-security-market-frameworks-review/2020/2020-notice-of-south-australia-inertia-requirements-and-shortfall.pdf?la=en

[11] National Grid. System operability framework. 2016. [Online]. Available from: https://www.nationalgrid.com/sites/default/files/documents/8589937803-SOF%202016%20-%20Full%20Interactive%20Document.pdf

[12] Garcia-Ruiz M, Cantos-Alcantara GJ, Martinez-Ramos JL, Marano-Marcolini A. Minimum Required Inertia for a Fully Renewable AC Interconnected System. In: 2019 International Conference on Smart Energy Systems and Technologies (SEST). 2019. p. 1–6. doi: 10.1109/sest.2019.8849019

[13] Zhang W, Wen Y, Chung CY. Inertia Security Evaluation and Application in Low-Inertia Power Systems. IEEE Trans Power Syst. 2025;40(2):1725–37. doi: 10.1109/tpwrs.2024.3420786

[14] Gu H, Yan R, Saha TK. Minimum Synchronous Inertia Requirement of Renewable Power Systems. IEEE Trans Power Syst. 2018;33(2):1533–43. doi: 10.1109/tpwrs.2017.2720621

[15] Zhang Z, Du E, Teng F, Zhang N, Kang C. Modeling Frequency Dynamics in Unit Commitment With a High Share of Renewable Energy. IEEE Trans Power Syst. 2020;35(6):4383–95. doi: 10.1109/tpwrs.2020.2996821

[16] Milano F, Dörfler F, Hug G, Hill DJ, Verbič G. Foundations and Challenges of Low-Inertia Systems (Invited Paper). In: 2018 Power Systems Computation Conference (PSCC). 2018. p. 1–25. doi: 10.23919/pscc.2018.8450880

[17] Technical specifications for grid-connected operation and control of electrochemical energy storage station— Part 7: Inertia support and damping control, DL/T 2246.7-2021. 2021.

[18] National Grid. Distribution Code: DC0079 frequency changes during large disturbances and their impact on the total system (Phase 4). 2019. [Online]. Available from: https://www.ofgem.gov.uk/sites/default/files/docs/2019/08/dc0079_d.pdf

[19] EirGrid and SONI. Mitigating technical challenges arising from high RES-E penetration on the island of Ireland. 2021. [Online]. Available from: https://www.soni.ltd.uk/media/documents/Technical-Assessmentof-2030-Study-Outcomes.pdf

[20] Zhao Z, Zhang K, Xue Y, Li Z, Tang Y, Wu Z. Generic Low-Order Primary Frequency Response Model for Frequency Nadir Prediction. In: 2024 China International Conference on Electricity Distribution (CICED), 2024. p. 946–52. doi: 10.1109/ciced63421.2024.10754573

[21] Garcia-Ruiz M, Cantos-Alcantara GJ, Martinez-Ramos JL, Marano-Marcolini A. Minimum Required Inertia for a Fully Renewable AC Interconnected System. In: 2019 International Conference on Smart Energy Systems and Technologies (SEST), 2019. p. 1–6. doi: 10.1109/sest.2019.8849019

[22] Egido I, Fernandez-Bernal F, Centeno P, Rouco L. Maximum Frequency Deviation Calculation in Small Isolated Power Systems. IEEE Trans Power Syst. 2009;24(4):1731–8. doi: 10.1109/tpwrs.2009.2030399

[23] Baldwin MS, Schenkel HS. Determination of frequency decay rates during periods of generation deficiency. IEEE Trans on Power Apparat Syst. 1976;95(1):26–36. doi: 10.1109/t-pas.1976.32074

[24] Ducoin EAS, Gu Y, Chaudhuri B, Green TC. Analytical Design of Contributions of Grid-Forming and Grid-Following Inverters to Frequency Stability. IEEE Trans Power Syst. 2024;39(5):6345–58. doi: 10.1109/tpwrs.2024.3351530

[25] Liu J, Miura Y, Ise T. Comparison of Dynamic Characteristics Between Virtual Synchronous Generator and Droop Control in Inverter-Based Distributed Generators. IEEE Trans Power Electron. 2016;31(5):3600–11. doi: 10.1109/tpel.2015.2465852

[26] Shintai T, Miura Y, Ise T. Reactive power control for load sharing with virtual synchronous generator control. In: Proceedings of The 7th International Power Electronics and Motion Control Conference, 2012. p. 846–53. doi: 10.1109/ipemc.2012.6258956

[27] Alipoor J, Miura Y, Ise T. Power System Stabilization Using Virtual Synchronous Generator With Alternating Moment of Inertia. IEEE J Emerg Sel Topics Power Electron. 2015;3(2):451–8. doi: 10.1109/jestpe.2014.2362530

[28] Wu Y, Chen Z, Chen R, Chen X, Zhao X, Yuan J, et al. Stochastic optimization for joint energy-reserve dispatch considering uncertain carbon emission. Renew Sustain Energy Rev. 2025;211:115297. doi: 10.1016/j.rser.2024.115297

[29] Peng Z, Peng Q, Zhang Y, Han H, Yin Y, Liu T. Online Inertia Allocation for Grid-Connected Renewable Energy Systems Based on Generic ASF Model Under Frequency Nadir Constraint. IEEE Trans Power Syst. 2024;39(1):1615–27. doi: 10.1109/tpwrs.2023.3267267

[30] Zhong Q-C. Virtual Synchronous Machines: A unified interface for grid integration. IEEE Power Electron Mag. 2016;3(4):18–27. doi: 10.1109/mpel.2016.2614906

[31] Anderson PM, Mirheydar M. A low-order system frequency response model. IEEE Trans Power Syst. 1990;5(3):720–9. doi: 10.1109/59.65898

[32] Cai G, Wang B, Yang D, Sun Z, Wang L. Inertia Estimation Based on Observed Electromechanical Oscillation Response for Power Systems. IEEE Trans Power Syst. 2019;34(6):4291–9. doi: 10.1109/tpwrs.2019.2914356

