# Supplementary material for: A new index for frequency stability assessment in Low-Inertia Power Systems
Source: PLoS One. 2026 Jan 7;21(1):e0340648. doi: 10.1371/journal.pone.0340648 (PMC12779145; doi:10.1371/journal.pone.0340648)
Supplement: S1 File — (DOCX) [file pone.0340648.s001.docx]

1.Minimal data set

URL：

https://zenodo.org/records/17705781?token=eyJhbGciOiJIUzUxMiJ9.eyJpZCI6IjA0OTkzY2RlLTBhZjAtNDZmNS1iNTQwLWQ4N2NlZDNhZjQ5NCIsImRhdGEiOnt9LCJyYW5kb20iOiI5YzUyOWZhZTIyYTlmOTA4ZGQwYTJjYjE5MTk2NGY1MCJ9.ee0Hh4uV8y136UxVlYV68Q0VfqhDFgT9bmzUIaXUBqJeqdijdwWxSL1PQwQxDAGgmc5IMMK11qJqtaS_jN0sug

DOI:

DOI [10.5281/zenodo.17705780](https://doi.org/10.5281/zenodo.17705780).

2.Sharing code

URL：

https://github.com/qin7616-ops/A-new-index-for-frequency-stability-assessment.git
